# Consumer Sentiments and Emotions in New Seafood Product Concept Development: A Co-Creation Approach Using Online Discussion Rooms in Croatia, Italy and Spain

**DOI:** 10.3390/foods12081729

**Published:** 2023-04-21

**Authors:** Marta Verza, Luca Camanzi, Cosimo Rota, Marija Cerjak, Luca Mulazzani, Giulio Malorgio

**Affiliations:** 1 Department of Agricultural and Food Sciences, Alma Mater Studiorum—Università di Bologna, 40127 Bologna, Italy; marta.verza2@unibo.it (M.V.); cosimo.rota@unibo.it (C.R.); luca.mulazzani@unibo.it (L.M.); giulio.malorgio@unibo.it (G.M.); 2 Faculty of Agriculture, University of Zagreb, 10000 Zagreb, Croatia; mcerjak@agr.hr

**Keywords:** seafood, new product development, co-creation, consumers’ acceptance, topic modeling, sentiment analysis, emotion analysis

## Abstract

Growing Mediterranean seafood consumption, increasing consumers’ awareness of food safety and quality, and changing food lifestyles are leading to the development of new food products. However, the majority of new food products launched on the market are expected to fail within the first year. One of the most effective ways to enhance new product success is by involving consumers during the first phases of New Product Development (NPD), using the so-called co-creation approach. Based on data collected through online discussion rooms, two new seafood product concepts—sardine fillets and sea burgers—were evaluated by a set of potential consumers in three Mediterranean countries—Italy, Spain, and Croatia. Textual information was analyzed by first using the topic modeling technique. Then, for each main topic identified, sentiment scores were calculated, followed by the identification of the main related emotions that were evoked. Overall, consumers seem to positively evaluate both proposed seafood product concepts, and three recurrent positive emotions (trust, anticipation, joy) were identified in relation to the main topics aroused during the discussions. The results of this study will be useful to guide future researchers and actors in this industry in the next development steps of the targeted seafood products in Mediterranean countries.

## 1. Introduction

Consumption of fish and seafood has been increasing in the European Union (EU) over the past years, especially in Mediterranean countries, where in 2019 the highest annual average seafood consumption was recorded (33.7 kg versus 20.7 kg per capita EU consumption) [1]. To satisfy the rapidly growing consumer demand in the Mediterranean area, the production of aquaculture and seafood products is expected to keep growing in the next years [2]. This, however, has come with alarming exploitation rates of fish stocks and unsustainable levels of wild fish catch, making overfishing one of the current biggest environmental problems in the Mediterranean Sea [3,4,5].

Given the significant contribution of the fishery sector to the livelihoods and food security of coastal populations in this region [4], finding innovative solutions to Mediterranean overfishing and promoting sustainable use and management of seafood is an imperative task for the long-term sustainability of this sector.

In this context, new product development (NPD) has the potential to enhance the long-term sustainability of marine resources and of the whole fishery industry by effectively combining technological innovation and market research. On the one hand, technological innovation allows the enhancement of product preparation, presentation, and shelf life to meet constantly rising standards [6]. On the other hand, market research has the potential to identify the most influential factors for seafood consumption, such as food safety, quality, price, and convenience [7,8,9,10,11,12,13,14]. Ready-to-eat or ready-to-cook food products are, indeed, more attractive to consumers than fresh and raw products [15]. Myrland et al. [16] found that the perception of fish as “difficult to prepare” negatively influences the purchase of whole fish. New convenient seafood products (e.g., burgers or ready-to-cook meals) can become an opportunity for both producers and retailers to attract consumers who normally are not seafood purchasers due to its long preparation time [17].

Although the current food market seems to largely cover most food categories [18], fewer new seafood products have been proposed compared with other food industries (e.g., meat), leaving room for NPD and exploration of potential consumers’ acceptance [19]. At the same time, few studies have analyzed consumers’ responses to new seafood product concepts, particularly in the Mediterranean area. Insights from such research could support the development of innovative food products and provide new successful and sustainable market opportunities for actors in the fishery industry.

To address this gap, we conducted a qualitative assessment of consumers’ attitudes (i) toward seafood in general and (ii) toward two innovative product concepts designed to allow for the sustainable exploitation of fish stocks and to improve their market acceptance. Considering the high likelihood of market failure in the NPD process, we chose to assess a priori consumers’ response to new food products through co-creation activities to develop a product design that meets the interests and needs of consumers [20,21]. The research was conducted in three major consumer markets, i.e., Croatia, Italy, and Spain [22,23,24].

Accordingly, the aim of this paper is twofold: (1) to identify the most relevant determinants affecting potential consumers’ acceptance of the proposed products in the Mediterranean area, and (2) to explore the usefulness of the proposed qualitative approach in generating new seafood product concepts.

## 2. Consumer Engagement in New Food Product Development

New product developments mostly originate from the evolution of new technologies or from new market opportunities. However, to avoid market failure of the new product, research shows one of the most effective ways to enhance new product success is based on the assessment of consumers’ first responses and attitudes [25].

**Figure 1:**
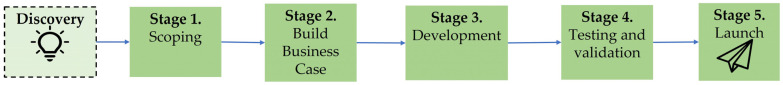
The Stage-gate progress, own illustration, adapted from Cooper [26].

In the literature, consumer-oriented studies are carried out along the process of NPD at five different stages: (1) scoping, (2) building a business case, (3) development, (4) testing and validation, and finally (5) market launch (Figure 1) [26]. This is referred to as the Stage-Gate process, a conceptual and operational system developed to move new products from idea generation to product launch in the NPD process [26].This process is made up of “stages”, i.e., investigation and development activities conducted throughout the NPD, and “gates”, where people involved in NPD discuss and decide on the progression or termination of new product ideas.

Other scholars, instead, reduced these steps to three main stages: (i) concept development, (ii) product development, and (iii) launch [27]. With a focus on the initial stage, concept development is defined as a stage that looks at generating novel ideas required to develop a new product. At this level, gathering customer information and feedback on new product concepts is fundamental to reducing costs in the later stages of the process and also decreasing the chance of product failure after launch [27,28].

Recently, research has shown that involving consumers in co-creation is a pivotal factor during the initial stages of NPD of idea generation, screening, and scoping [19,28,29,30]. Co-creation has been defined as a process that directly involves different types of stakeholders, including consumers, in idea generation [19]. The collaboration between developers and customers through co-creation not only brings value creation in product development [11] but also explores consumers’ wants and needs [30]. In this regard, we should remark on the work by Banovic et al. [29], who demonstrated that involving consumers in the process of new food product development through co-creation is extremely useful to gain actionable insights for the future marketing of new fish products.

At the first stage of scoping and concept development, qualitative research seems to be an appropriate approach to understanding how consumers perceive new product concepts through feedback and comments. Specifically, data obtained through consumers’ direct involvement in NPD are often used to gain insights into consumers’ attitudes and consumption choices to generate new products aligned as much as possible with their needs and desires. In the literature, this can be achieved through the use of focus groups [31,32] as well as through sensory analysis and web-based experiments [30,33].

In light of this, the paper focuses on the initial stage of the NPD process, namely understanding consumers’ responses and attitudes related to the first ideation of new seafood product concepts through co-creation with potential consumers.

## 3. Materials and Methods

### 3.1. Product Concepts

The co-creation process for the development of new product concepts focused on two innovative seafood products: sardine fillets and sea burgers.

The two seafood product concepts were selected based on a set of criteria, agreed upon with an international panel of stakeholders from Croatia and Italy, including research agencies, producer organizations, and regional public authorities:

- Current favorable sustainability conditions of Adriatic fish species and potential for their exploitation;
- Potential for the processing innovation uptake of the new product concepts;
- Potential for their value creation and market penetration.

Specifically, in this identification process, sardines were considered suitable for sustainable exploitation while allowing for processing development toward an extended shelf life. On the other hand, sea burgers appear to have a high market potential, as a ready-to-cook or ready-to-eat solution, responding to the needs of modern consumers for more convenient food [19].

Both products were presented to the participants in the discussion rooms with a brief description of each concept at the beginning of the related discussion (Table 1).

### 3.2. Participant Recruitment

Due to the COVID-19 pandemic, online discussion rooms (two per country) were conducted to collect data in each of the three Euro-Mediterranean target countries, i.e., Croatia, Italy, and Spain.

Data were collected from the 22 to the 26 June 2020 in Croatia, Italy, and Spain, and each of the discussion rooms lasted for a total of 5 days. Participant recruitment took place through a market research agency specialized in multi-country panel surveys. Recruited respondents were awarded by the agency with incentives as a way to encourage active participation for the entire time of data collection.

In this study, participation was voluntary and anonymous; hence, no personal information was gathered in this study.

The benefits of using discussion rooms include the possibility to directly involve potential consumers, to construct new knowledge as individuals freely share information through online conversations, and the time lag between postings, which leads to more reflective responses than those of face-to-face situations. It is proven that this technique allows a clear view of participants’ discussions without the presence of the researcher possibly influencing their response [34].

The country selection was based on the importance of Mediterranean countries in European seafood production and consumption and on the results of a report by EUMOFA [1]. In this report, Spain was found to have the second-highest annual consumption of seafood in Europe while Italy was positioned among the ten highest-consuming EU countries. In the case of Croatia, although its consumption remained below the European average, an interesting trend toward increasing consumption of fish and seafood (+9%) is observed [1].

In total 191 participants joined the discussion rooms—47 in Croatia, 65 in Italy, and 79 in Spain.

### 3.3. Online Discussion Rooms

Each online discussion room was animated by a moderator. Moderators were professionals from the agency involved in the research. Standardized protocols were used by all moderators to share common guidelines and targeted questions aiming at encouraging the constant active participation of the respondents.

Specifically, the moderators followed a detailed discussion guide that included three sections (Table 2):

- A warm-up debate about participants’ consumption, dietary, and cooking habits with regard to seafood, followed by a brief discussion on fish and fish product sustainability to gain a deeper understanding of people’s awareness and perceptions of this sector.
- A second section where participants discussed their first impressions, opinions, perceptions, and attitudes toward the first product concept—i.e., sardine fillets.
- A third section where participants discussed their first impressions, opinions, perceptions, and attitudes toward the second product concept—i.e., sea burgers.

The first theme introduces the concept of seafood consumption, participants’ dietary and cooking habits, as well as fish sustainability and participants’ related perceptions. After a discussion on these general topics, participants are introduced to two new product concepts: sardine fillets (Theme 2) and sea burgers (Theme 3), on which the moderator guided the discussion around to evoke consumers’ reactions and perceptions.

The discussions were conducted in the native language of each country and then translated into English and transcribed verbatim for further analysis.

### 3.4. Data Analysis

**Figure 2:**
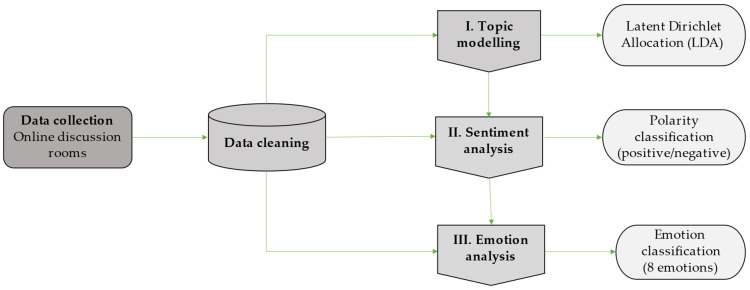
Data collection and processing outline, own elaboration.

The textual information collected was preliminarily cleaned, removing from the text any emojis, special characters, punctuation marks, or typing errors [35] (Figure 2).

The following data analysis entailed three main steps: (I) main topic identification through topic modeling, (II) polarity classification of the identified topics through sentiment analysis, and (III) emotion evaluation of topics related to the new product concepts (Figure 2).

#### 3.4.1. Topic Modeling

With the increasing availability of automatic computational capacities, topic modeling has been used as an efficient, computer-assisted technique for analyzing textual content [35,36,37]. Topic models are especially useful for identifying hidden topics based on the word co-occurrence in the corpus of a document. To do that, in this paper, LDA (Latent Dirichlet allocation) topic modeling was applied [38]. The LDA is an algorithm used to detect topics and their distribution by the means of the term frequency [37]. To apply topic modeling in this research, the MALLET (MAchine Learning for LanguagE Toolkit) Java-based package was used [39]. It is said that the number of topics to detect in LDA greatly impacts the validity and interpretability of the results; if the number of topics is too small, the resulting topics may be too broad and heterogeneous, while if the number of topics is too large, the topics may be overly specific and difficult to interpret [40].

The number of topics to detect relates to two parameters: (i) the alpha parameter, which defines the prior on the per-document topic distributions and controls the weight of each topic in a document, and (ii) the beta parameter, which define the prior on per-topic multinomial distribution over words and controls the weight of each word in a topic [41]. In general, a common approach is to set the alpha parameter to a small value (less than 1), which favors sparse topic distributions in documents. Similarly, the beta parameter is often set to a very small value (much less than 1), which also favors sparse word distributions within topics.

For the purpose of this study, the values settled for alpha and beta parameters in topic modeling are, respectively, 0.1 and 0.01. Ultimately, in order to evaluate the coherence and interpretability of the resulting topics, the domain knowledge approach was used [42]. Examining topics and topic-term distributions generated by the analysis, it was possible to identify topics consistent with the subject matter and identify any topics that seem irrelevant or unclear.

#### 3.4.2. Sentiment Analysis

After topic modeling, sentiment analysis was performed. Sentiment analysis, also known as opinion mining, is a type of analysis used to extract and classify the polarity of a given text/sentence in a document (positive, negative, or neutral) in relation to the use or perception of products or services [36]. Sentiment analysis is generally used to discover the general public’s hidden opinions or knowledge from text data and applied simultaneously in different fields, such as behavioral science, political science, or social science [36].

The sentences extracted from the discussion room chat, examined using the R software and its associated library, the NRC Emotion Lexicon, are classified as positive, negative, or neutral according to a score range indicating how negative or positive the text analyzed is [43]. The exact threshold for what is considered positive may depend on the specific implementation or use case, but in general, sentiment scores closer to 1 indicate a higher level of positive sentiment [43]. According to the adopted Text Analytics Library by Microsoft Azure Cognitive Service [44], an input text that is mostly neutral results in a 0.5 score.

For the purpose of this study, scores above 0.65 were considered positive.

#### 3.4.3. Emotion Analysis

According to Farkhod et al. [36], advanced sentiment classification, also called “beyond polarity” classification, can additionally look at the emotional states of the identified topics. Based on the work by the psychologist Robert Plutchik [45], eight basic emotions can be identified: two positive (joy and trust), two ambivalent (surprise and anticipation), and four rather negative (sadness, fear, disgust, and anger). Emotion detection is based on this classification, and the output from this type of analysis shows how many occurrences of words associated with that particular emotion exist in that line of the text.

The analysis was carried out using the R software and the NRC Emotion Lexicon to classify the proportion of words and sentences that indicate certain emotions.

## 4. Results

Topics and sentiment results are presented in three separate subsections, one per each theme of discussion (see Table 1), and are displayed in tables reporting the identification number and the key related terms for each topic with the highest sentiment scores. In addition, a label was assigned to each topic to categorize the content using keywords and to make the interpretation of results easier and more straightforward. Finally, the main representative chat texts for each topic were extracted from the database for a better exemplification of the topic’s meaning.

Results from the emotion analysis are also presented in two separate subsections, one per each theme of discussion (see Table 1). In this study, visualization with tables was preferred to better show value numbers and percentages of emotion occurrences [46].

In the body text of this study, the main results are displayed while full detailed results for both sentiment and emotion analysis can be found in the Supplementary material (Tables S1–S12).

### 4.1. Theme 1: Attitudes toward Seafood

#### 4.1.1. Topic Modeling and Sentiment Analysis Results

The elaborations on Theme 1 regarding consumers’ attitudes toward seafood resulted in five main topics, ranked with sentiment scores ranging from 0.66 to 0.78 (Table 3).

Specifically, topic_4, labeled as “Habitual consumption”, has the highest score (0.78), followed by “Cooking” (topic_2) with a sentiment score of 0.73 (Table 3). Based on these results, it seems that most participants have a highly positive attitude toward preparing, eating, and cooking seafood products in their daily life.

“Freshness” of seafood products (topic_1) is also found to be a critical determinant of seafood consumption and was positively evaluated in all the countries under investigation (0.71) (Table 3). Finally, attitudes toward sustainability linked with both packaging (topic_3) and fishing (topic_0) were assessed. Overall, positive scores are calculated for these two topics—respectively, 0.67 and 0.66 (Table 3).

At the country level, Italians stand out as the respondents with the highest positive sentiment toward cooking seafood (0.86), specifically toward already-cleaned fish and specific fish species such as mussels (Table S1). Similarly, Croatians also appear to have a positive attitude toward preparing fish in their daily life (0.81) and buying fresh fish (0.74) (Table S3) while Spanish participants seem to have a highly positive attitude toward buying fresh produce (0.73) and have specific cooking preferences toward fish such as hake and prawns (Table S2).

Additional findings on fish and fishing sustainability show that the two countries with the highest positive score are Croatia (0.71) (Table S3) and Italy (0.71) (Table S1). Finally, the perception of fish as “difficult to prepare” is found to be another aspect of discussion among participants. Specifically, the Spanish discussed aspects surrounding the “time” of fish preparation (topic_3) (Table S2) while in Croatia, participants argued around aspects such as the “smell” and “long cleaning preparation time” (topic_3) (Table S3).

#### 4.1.2. Emotion Analysis Results

Based on the previous topic modeling, we analyzed the most frequent emotions expressed by the participants in correspondence with the identified topics.

In Table 4, we present the most recurrent emotions surrounding attitudes toward seafood (Theme 1) across all countries. As seen in Table 3, in total, 1,045 chat texts were analyzed in this theme.

The first most recurrent positive emotion is *trust* and is found mainly in relation to the topic “Sustainable fish and fishing”. Out of 185 analyzed chat texts on this topic (Table 3), trust emerges 223 times (120.5%) (Table 4). A rather high number of emotion occurrences for trust is also found in the topic “Cooking”. Out of the 116 chat texts analyzed on this topic (Table 3), trust appears 124 times (106.9%) (Table 4). *Trust* is finally found to be a recurrent emotion also in relation to “Sustainable packaging”, where out of 163 analyzed chat texts on this topic (Table 3), this emotion emerges 112 times (68.7%) (Table 4).

The second most recurrent positive emotion is anticipation and is mostly found in relation to the topic “Habitual consumption”. Out of the 249 chat texts analyzed on this topic (Table 3), anticipation appears 288 times (115%) (Table 4).

Finally, joy is the third most recurrent positive emotion and is mainly found in relation to the topic “Cooking”. Out of the 116 analyzed chat texts on this topic (Table 3), *joy* appears 102 times (87.9%) (Table 4).

### 4.2. Theme 2: Sardine Fillets

#### 4.2.1. Topic Modeling and Sentiment Analysis Results

Table 5 illustrates the results of the topic detection ordered from the most positive sentiment scores on words associated with the new sardine fillets concept.

Overall, across all three countries, the predominant sentiment associated with this product seems to be positive. Based on the product description provided to the participants, people seem to perceive the sardine concept as healthy (topic_2) (0.87) and practical to consume (topic_0) (0.75) (Table 5). The second most positively evaluated aspect appears to be price (topic_1) (0.76), suggesting that in these countries, respondents believe price is one of the most important determinants for their seafood consumption behavior.

However, at the country level, participants seem to pay attention to other sardine product characteristics. For instance, Croatians have a high positive sentiment associated with both its prolonged shelf life (0.91) and its convenience (0.86) (Table S6) while Italian respondents seem to appreciate more product characteristics such as quality (0.88) and freshness (0.86) (Table S4). As in Croatia, participants in Spain seem also to give high importance to aspects such shelf life of the product (0.85) while showing a high positive willingness to try or buy sardine fillets (0.79) (Table S5).

Overall, healthiness, price, and convenience seem to be the three most critical determinants for consumers of the investigated countries to accept the new sardine fillets product. Participants seem also to positively appreciate the prolonged shelf-life characteristic of this innovative product concept.

#### 4.2.2. Emotion Analysis Results

According to Table 6, the most recurrent emotions experienced by participants in relation to the sardine fillets concept (Theme 2) are positive across all three countries of investigation. As seen in Table 5, in total, 549 chat texts were analyzed in this theme.

The first most recurrent positive emotion is anticipation and is found mainly in relation to the topic “Healthiness”. Out of the 128 analyzed chat texts on this topic (Table 5), anticipation is found 193 times (150.8%) (Table 6). A fairly high number of emotion occurrences for anticipation is also found in the topic “Price”, where out of 178 analyzed chat texts on this topic, this emotion appears 206 times (115.7%) (Table 6).

The second most recurrent positive emotion is *trust.* This emotion is mainly found in relation to “Healthiness”, emerging in this topic 175 times (136.7%) (Table 6). In terms of occurrences, trust also appears in relation to the topic of “Species”. Out of 110 analyzed chat texts on this topic, trust appears 94 times (85.5%) followed by the topic “Convenience”, where out of the 133 analyzed chat texts (Table 5), trust emerges 112 times (84.2%) (Table 6).

Finally, the third most positive emotion is joy, mainly found in relation to the “Healthiness” of the proposed sardine fillets. Out of the 128 analyzed chat texts on this topic (Table 5), joy appears 127 times (99.2%) (Table 6).

### 4.3. Theme 3: Sea Burgers

#### 4.3.1. Topic Modeling and Sentiment Analysis Results

As shown in Table 7, across all analyzed countries, participants seem to express a positive sentiment associated with this new product concept too.

The highest positive score (0.85) associated with this product is found in the perception of eating the sea burger raw (topic_4). Considering the description provided to participants on the HHP treatment technology applied to this product, respondents seem to express a positive attitude toward the potential consumption of raw sea burgers.

Furthermore, the majority of respondents across the three countries of investigation also express a highly positive (0.76) willingness to buy or eat (topic_0) the proposed product. Participants also expressed ideas on the most preferred eating time for this innovative product (topic_2) (Table 7). Based on the chat text example of this topic, one participant suggests that, given it is a practical, ready-to-cook product, this burger would be ideal for dinner time, when consumers generally have no time or ideas for food preparation.

Finally, participants also discussed preferred ingredient formulations and related consumption habits. More specifically, most respondents seem to have a high positive sentiment (0.71) toward this burger if it was made of shrimps or prawns (topic_3) (Table 7).

At the country level, Italian respondents appear to be the ones with the highest positive registered score (0.96) associated with the potential consumption of this burger raw (topic_2) (Table S7) followed by Croatians with a registered score of 0.90 (topic_1) (Table S9). Furthermore, Croatian respondents stand out as the country with the highest willingness to try or eat this product (0.96) (Table S9) while Spanish participants discussed their preferences for the species composition of the burgers more, expressing a positive preference toward prawn-based burgers (topic_1) (Table S8).

#### 4.3.2. Emotion Analysis Results

Based on Table 8, it can be noticed that the most recurrent emotions experienced by participants in relation to the sea burger concept (Theme 3) are positive across all three countries of investigation. As seen in Table 7, in total, 591 chat texts were analyzed in this theme.

The first most recurrent positive emotion is *anticipation* and is found mainly in relation to “Willingness to buy/eat”. Out of the 86 analyzed chat texts on this topic (Table 7), anticipation appears 112 times (130.2%) (Table 8).

Secondly, trust is also found to be one of the most recurrent positive emotions, appearing 90 times (104.7%) in the topic “Willingness to buy/eat” (Table 8). The *trust* emotion is also found in relation to the possibility of eating the sea burger raw (topic_4 “Raw fish”). Specifically, out of 114 analyzed chat texts on this topic, trust emerges 113 times (99.1%) (Table 8).

Finally, joy is the third most recurrent emotion that emerged in the discussions on the sea burger concept. This emotion is aroused mostly in relation to the “Willingness to buy/eat” the proposed product with an occurrence of 81 times (94.2%). The emotion joy also appears in relation to eating the sea burger raw (topic_4 “Raw fish”) with an occurrence of 90 times (78.9%) (Table 8).

## 5. Discussion

In this present study, the co-creation process with potential consumers identified the main factors influencing their potential acceptance of the new seafood product concepts as well as sentiments and emotions aroused in relation to the proposed products.

The analysis showed overlapping influencing factors among the three targeted countries, suggesting that seafood consumption behavior in the Mediterranean area may share common consumption traits. The information collected will help to advance research on public attitudes and evoked sentiments and emotions toward the development of new seafood products.

Results from the present study indicate that “convenience” was one of the leading drivers of positive consumer attitude toward both new seafood product concepts, as this directly led to reflection about time preparation and safe ready-to-eat or cook solutions. This is in line with previous literature, where convenience was repeatedly found as an important factor to expand general seafood consumption [47,48,49]. This further validates that the issue of convenience is critical in encouraging consumers in buying or eating new seafood products, representing a concrete opportunity to reach consumers who normally do not buy seafood due to its smell and long preparation time [15,17,50]. Specifically, Italians registered one of the highest positive sentiment scores toward the newly developed sea burger concept. This is in line with the findings from a study by Spada et al. [51], who also found a positive attitude toward newly developed fish-based burgers in Italy. Findings recorded in this study and in the previous literature may imply that newly developed sea burgers may have a good chance of success once launched in the targeted market.

In addition, “shelf life” was also found to be an important influencing factor for seafood consumers in the present study. Specifically, since the start of the COVID-19 pandemic, consumers have been more concerned about food safety in the fishery sector [52]. The increased concern about food safety has directly contributed to greater consumer acceptance of new products developed using innovative packaging technologies that extend a product’s shelf life and improve its food safety characteristics [53].

At the country level, Croatians seem to be more concerned about aspects such as shelf life and convenience of the seafood product concepts while Italian respondents seem to appreciate more characteristics such as quality and freshness. Similar to Croatians, Spanish participants positively evaluate the longer shelf-life characteristics while expressing specific preferences about the species composition in the development of the burger product concept.

The results also support the effectiveness of emotion analysis as a qualitative technique to facilitate public perceptions of new seafood product concepts. In particular, participants expressed positive emotions in relation to all three themes of discussion. Most respondents show a high positive level of trust toward the topic of fish and fishing sustainability. This is in line with previous findings revealing an overall consensus about a positive consumer preference toward sustainable fish [13], thus emphasizing that sustainable aspects are important factors to consider in the marketing of new seafood products. Trust was also found in relation to the perceived healthiness of the sardine fillet products. This is supported by previous studies which found that, generally speaking, fish is positively perceived as a healthy food [54]. Other positive emotions such as joy were found in relation to seafood cooking activities, suggesting that targeted consumers generally enjoy preparing and experimenting with seafood meals at home.

### 5.1. Implications for Practice

Firstly, to improve the development of new products developed with new packaging technologies, the government should support scientific research toward NPD in the fishery sector, creating more economic opportunities and collaboration between science and the fishery industry to achieve a transformative change toward new sustainable fish market opportunities. Secondly, to improve consumers’ trust in these innovations, the government should promote and popularize knowledge about and the contribution of new seafood products developed with innovative packaging technologies.

### 5.2. Limitations and Future Research Directions

All in all, the information gathered might help researchers and marketers to guide or improve the future design of new seafood products in the Croatian, Italian, and Spanish markets.

In future research, assuming factors of consumers’ statistical characteristics as control variables, such as age, gender, education level, and income, can further explore the differences in consumers’ perceptions and emotional characteristics of newly developing products under different sample dimensions.

Moreover, as already highlighted by López-Masco et al. [19], co-creation with consumers during the first phases of NPD has some possible limitations. Consumers might generate ideas based on their own personal preferences rather than considering social or environmental issues [55]. Additionally, being a hypothetical assessment, consumers may generate a product idea that later may not match their actual purchasing behavior [19].

## 6. Conclusions

Understanding consumers’ attitudes, sentiments, and emotions toward new seafood products is of great value for developing sustainable innovation in the fishery industry and answering to specific new consumption trends. This study overcomes certain limitations of traditional scientific research in terms of data acquisition (e.g., during the COVID-19 pandemic) by using online discussion rooms to explore consumers’ attitudes and emotional tendencies toward new seafood product concepts. The results show that consumers’ concerns about the newly proposed products follow a significant positive trend. Overall, the majority of targeted consumers are concerned about healthiness, convenience, and shelf life of the newly developing seafood product concepts, meanwhile trust, anticipation, and joy are found to be the main positive emotions aroused in all three investigated countries. Results from this study reveal that consumers’ feedback and perceptions could provide a great level of assistance to the development of new seafood products matching recent new consumer concerns while providing new successful market opportunities in the seafood industry.

## Figures and Tables

**Table 1:** Product concept descriptions.

Product Concept	Product Description
Sardine fillets	Sardine fillets packaged in a modified atmosphere (MAP) and presented in trays with cling film. 200 g, 2–3 servings. Preserved at a temperature between 1° and 4° C, their conservation is prolonged from 8 days, the approximate life of a conventional product, to 12 days (+50%) maintaining its stable quality characteristics (e.g., microbiological aspects, color, etc.).
Sea burgers	Sea burgers made of white fish and crustaceans (e.g., mullets and shrimps) vacuum packed under high hydrostatic pressure (HHP) and presented in trays with cling film. 500 g, 2–3 servings. Preserved at a temperature between 1 and 4 °C, their conservation is prolonged from about 5 days, typical of a conventional product, up to 30 days while maintaining their stable quality characteristics (e.g., microbiological aspects, color, etc.). Furthermore, within 7 days of the HHP treatment, the burger can be eaten raw (as a ready-to-eat tartare).

**Table 2:** Discussion themes and related stimuli.

Themes	Stimuli for Discussion
1. Attitudes toward seafood	Where do you usually buy/eat sea fish? What are the most important features of the seafood you buy/eat? On which occasions do you usually eat seafood? Who prepares it? How is it prepared? What do we mean by “sustainable seafood”? What signs can tell you that a seafood product is “sustainable”?
2. Product concept A— Sardine fillets 3. Product concept B— Sea burgers	Product A/B description. What are the first three adjectives that came to your mind to describe this product? What are your first impressions (positive or negative) about the product? On which occasions would you like to eat this product? How does it compare with other similar products already existing on the market? How likely are you to buy it?

**Table 3:** Theme 1 topic and sentiment scores_ALL COUNTRIES.

Topic ID	Key Terms	Topic Label	Overall Sentiment	Number of Chat Texts	Chat Text
topic_4	fish, prepare, usually, eat	Habitual consumption	0.78	249	“I usually eat seafood with my family or in a restaurant. Depending on the recipe, I prepare the dishes myself with the help of other members. Sometimes I like to create new recipes; sometimes I enjoy traditional recipes.”
topic_2	fish, sea, oil, garlic	Cooking	0.73	116	“I usually cook bigger fish, like sea bream. We do not like it too complicated; we just season it a little and serve it with good homemade olive oil, parsley, and onions (garlic).”
topic_1	fish, buy, fresh, usually	Freshness	0.71	332	“We buy seafood from local fishermen and at the fish market, from acquaintances who recommend what fish to buy. The most important thing is that it is fresh.”
topic_3	packaging, fish, product, sustainability	Sustainable packaging	0.67	163	“In my opinion, there is a link between sustainability and the packaging of fish. Even the packaging must be sustainable; it must be disposed of without polluting the environment, perhaps using biodegradable plastic.”
topic_0	fish, sustainable, fishing, sea	Sustainable fish and fishing	0.66	185	“Fishing must be done in a sustainable way, without depleting the fisheries and guaranteeing the future of the species. We must all get involved in the fight to use respectful fishing gear and avoid depleting species.”
**Total chat texts**				**1.045**	

**Table 4:** Theme 1 Main emotion results with % value and absolute value_ALL COUNTRIES.

	Cooking		Habitual Consumption		Sustainable Fish and Fishing		Freshness		Sustainable Packaging	
Emotions	% on texts	emotion_ count	% on texts	emotion_ count	% on texts	emotion _count	% on texts	emotion_ count	% on texts	emotion_ count
anticipation	100.0%	116	115.7%	288	49.2%	91	22.9%	76	38.0%	62
joy	87.9%	102	82.7%	206	38.4%	71	18.7%	62	38.0%	62
trust	106.9%	124	87.6%	218	120.5%	223	52.4%	174	68.7%	112

**Table 5:** Theme 2 topics and sentiment scores_ALL COUNTRIES.

Topic ID	Key Terms	Topic Label	Overall Sentiment	Number of Chat Texts	Chat Text
topic_2	product, healthy, description, fish	Healthiness	0.87	128	“Fast, healthy, delicious. It struck me that I would consume more fish. The product description is understandable. I like it.”
topic_1	product, buy, price, fish	Price	0.76	178	“Yes, the price will also be important, because in order to eat with that, even with two at home, you have to buy several packages!”
topic_0	product, fish, loins, time	Convenience	0.75	133	“I consider the loins to be more practical when it comes to cooking because they are more versatile than using whole fish.”
topic_3	sardines, anchovies, eat, fish	Species	0.73	110	“Anchovies seem very strong to me and I much prefer clean sardines.”
**Total chat texts**				**549**	

**Table 6:** Theme 2 Main emotion results with % value and absolute value_ALL COUNTRIES.

	Convenience		Healthiness		Price		Species	
Emotions	% on texts	emotion_ count	% on texts	emotion_ count	% on texts	emotion_ count	% on texts	emotion_ count
anticipation	56.40%	75	150.80%	193	115.70%	206	50.90%	56
joy	49.60%	66	99.20%	127	57.30%	102	48.20%	53
trust	84.20%	112	136.70%	175	75.30%	134	85.50%	94

**Table 7:** Theme 3 topics and sentiment scores_ALL COUNTRIES.

Topic ID	Key Terms	Topic Label	Overall Sentiment	Number of Chat Texts	Chat Text
topic_4	fish, product, raw, burger	Raw fish	0.85	114	“The word burger automatically puts me off, but if it involves fish and shrimp, then it’s okay. I’d still give it a try. I like fish stored in a more traditional way. It looks more natural, nicer. I would eat a raw burger because I do like that when the appropriate storage rules are adopted.”
topic_0	product, fish, buy, eat	Willingness to buy/eat	0.76	86	“I tried the tuna burgers; they were brilliant for me. I like the idea. I would definitely try it. If I had to say something to a friend about the product, I’d probably say if you’re in the mood for fish and do want to pay that much for a fish, buy these fish burgers”
topic_2	product, dinner, fish, consume	Eating time	0.76	173	“The most appropriate time to consume it I think will be at night during dinner since it is an ideal product to cook when you have no idea about what to prepare at that time.”
topic_3	fish, shrimp, prawns, prefer	Species	0.71	128	“On whether prawns or shrimp, much better prawns for my taste, they are less strong.”
**Total chat texts**				**591**	

**Table 8:** Theme 3 Main emotion results with % value and absolute value_ALL COUNTRIES.

	Species		Eating Time		Raw Fish		Willingness to Buy/Eat	
Emotions	% on texts	emotion_ count	% on texts	emotion_ count	% on texts	emotion_ count	% on texts	emotion_ count
anticipation	29.70%	38	72.80%	126	91.20%	104	130.20%	112
joy	27.30%	35	43.40%	75	78.90%	90	94.20%	81
trust	53.10%	68	67.60%	117	99.10%	113	104.70%	90

## Data Availability

The data presented in this study are available on request from the corresponding author (L.C.).

## Supplementary Materials

The following supporting information can be downloaded at: https://www.mdpi.com/article/10.3390/foods12081729/s1, Table S1: Theme 1 Topics and sentiment scores_ITALY; Table S2: Theme 1 Topics and sentiment scores_SPAIN; Table S3: Theme 1 Topics and sentiment scores_CROATIA; Table S4: Theme 2 Topics and sentiment scores_ITALY; Table S5: Theme 2 Topics and sentiment scores_SPAIN; Table S6: Theme 2 Topics and sentiment scores_CROATIA; Table S7: Theme 3 Topics and sentiment scores_ITALY; Table S8: Theme 3 Topics and sentiment scores_SPAIN; Table S9: Theme 3 Topics and sentiment scores_CROATIA; Table S10: Theme 1 Emotion results with % value and absolute value_ALL COUNTRIES; Table S11: Theme 2 Emotion results with % value and absolute value_ALL COUNTRIES; Table S12: Theme 3 Emotion results with % value and absolute value_ALL COUNTRIES.
