# Supplementary material for: Consumer Sentiments and Emotions in New Seafood Product Concept Development: A Co-Creation Approach Using Online Discussion Rooms in Croatia, Italy and Spain
Source: Foods. 2023 Apr 21;12(8):1729. doi: 10.3390/foods12081729 (PMC10138044; doi:10.3390/foods12081729)
Supplement: Supplementary file 1 [file foods-12-01729-s001.zip › foods-2315283-supplementary.pdf]

foods

MDPI

Supplementary material

Table S1 Theme 1 Topics and sentiment scores_ ITALY

Topic id	Key Terms	Topic label	Overall sentiment	Number of chat texts	Chat text
topic_0	fish, sea, cook, oil	Cooking	0.86	49	*With fish I use a lot of garlic, lemon and oil. Usually in the evening I use to make fish in foil, while for lunch I make first and second courses based on fish.*
topic_1	fish, clean, understand, mussels	Preparation mode	0.73	29	*If the fish is fresh, it needs very few ingredients and very simple cooking. As for the type of fish to prepare, the decision is linked to the fact that my children do not know how to clean it themselves, so we prefer fish that are not very small and easy to bone.*
topic_2	fish, agree, fresh, course	Freshness of the fish	0.72	38	*I buy the fish at the supermarket or at the fish market, under the gills there must be a bright red, the eye must be crystalline and white, it should scent of sea, but it must not smell; I buy fish with the head if possible and with the head I make the sauce for the first course.*
topic_4	fish, buy, fresh, freshness	Freshness in purchase	0.72	69	*I usually buy fish in the fish shop near the house, where freshness is guaranteed, a couple of times a week, but it happens that I also get something at the supermarket, in this case frozen.*
topic_3	fish, fishing, sustainable, sea	Sustainable fish and fishing	0.71	61	*Sustainable fish is certainly fish that is caught with techniques that do not "harm" the species and in a way that respects the sea. In short, a fishing that does not cause irrecoverable damage to the ecosystem.*
**Total chat texts**				**246**	

Table S2 Theme 1 Topics and sentiment scores_SPAIN

Topic id	Key Terms	Topic label	Overall sentiment	Number of chat texts	Chat text
topic_4	fish, usually, eat, hake	Consumption	0.80	93	*At home we like to eat fish at least twice a week, it is a natural and very healthy food. In particular, I do like fish with strong flavors, so I prefer hake, sole or sea bass.*
topic_1	fish, buy, usually, fresh	Freshness in purchase	0.73	124	*We buy the fish in the food market, the most important thing for us is that it be quality fish and above all that it is fresh.*
topic_2	fish, cook, hake, prawns	Cooking	0.70	43	*We cook them both myself and my husband, and I accompany them with salads, sauces and a fideua or paella and we consume squid, sardines, anchovies, salmon, prawns, hake, etc.*
topic_0	fish, frozen, prefer, fresh	Frozen preference	0.65	75	*I prefer frozen fish because from what I understand it is the only way to avoid being infected with anisakis among other parasites.*
topic_3	time, fish, buy, price	Time preparation	0.59	56	*I do take into account the difficulty of preparation. I usually buy fish that is easily prepared, to avoid spending too much time deboning.*
**Total chat texts**				**465**	

Table S3 Theme 1 Topics and sentiment scores_ CROATIA

Topic id	Key Terms	Topic label	Overall sentiment	Number of chat texts	Chat text
topic_0	fish, prepare, usually, sea	Consumption	0.81	89	*I buy seafood at the fish market. Not often, but when I buy fish, it is important to me that it is not frozen and that it is from our sea. In rare situations, I buy packaged and frozen because I like to cook and prepare meals.*
topic_1	fish, buy, fresh, seafood	Freshness in purchase	0.74	72	*When buying,* it is *important to me that the fish / marine organism is fresh and not (in some species) tough or tasteless.*
topic_2	sustainable, product, fish, seafood	Sustainable fish	0.71	106	*Sustainable fishing leads to a significant recovery of fish habitats and is key to preserving the environment. It is certainly important to support sustainable products that are caught in a way that sustains fish species.*
topic_3	fish, smell, complicated, cleaning	Preparation mode	0.63	67	*It's not complicated cleaning fish for me because mostly I don't prepare it. Sometimes I just mind the smell of fish during baking.*
**Total chat texts**				**334**	

Table S4 Theme 2 Topics and sentiment scores_ITALY

Topic id	Key Terms	Topic label	Overall sentiment	Number of chat texts	Chat text
topic_3	product, quality, description, freshness	Product attributes perceptions	0.88	20	*The first three adjectives that come to my mind are: fresh – natural - superior. Because from how it is described it is something better in quality than the product that we usually find for sale in this category.*
topic_5	product, fish, fresh, idea	Freshness of the fish	0.86	32	*Fresh, tasty and convenient. The description is clear and the fact that they are sold on a tray gives me the idea of a fresh and safe product.*
topic_4	product, buy, time, dinner	Willingness to buy	0.80	40	*I would definitely buy it because it is a product that can be useful for preparing a quick meal, perhaps if you have little time available.*
topic_0	product, fish, time, cook	Time preparation	0.78	27	*To me this product seems ideal for a quick dinner, when you do have much time to devote to the preparation of something more elaborate.*
topic_1	lunch, dinner, buy, eat	Eating time	0.76	23	*I would consume it as a quick side dish or for a light dinner*
**Total chat texts**				**174**	

Table S5 Theme 2 Topics and sentiment scores_ SPAIN

Topic id	Key Terms	Topic label	Overall sentiment	Number of chat texts	Chat text
topic_0	product, time, fish, days	Shelf-life	0.85	73	*The product seems great to me, and the durability has surprised me that it is so much, but if it is good and suitable for consumption after 5 days, very good because it will help sustainable fishing.*
topic_1	product, fish, try, buy	Willingness to try/buy	0.79	42	*They are ideal for fast foods because they are made in a short time. And I would be very interested to try it and then decide whether to keep buying it or not.*
topic_2	anchovies, sardines, buy, price	Willingness to pay	0.78	55	*I would definitely buy it but I think I would not pay more than 7.50 euros for a 200 g container.*
topic_3	sardines, loins, product, fish	Product attributes perceptions	0.75	42	*Sardine loins packed in atmosphere look very good to me. The description is understandable and very well understood. As adjectives I would define it as a ready-to- eat product, preserving its best characteristics and the product is sustainable.*
topic_4	price, consume, product, buy	Price	0.67	87	*Fish can be consumed at any time and in any situation, or at least I do. The only thing is that I buy fish at affordable prices in my pocket, although sometimes I indulge myself.*
**Total chat texts**				**299**	

Table S6 Theme 2 Topics and sentiment scores_ CROATIA

Topic id	Key Terms	Topic label	Overall sentiment	Number of chat texts	Chat text
topic_3	product, description, life, shelf	Shelf-life	0.91	13	*I would describe this product as safe, high quality, and easy to use because it is already filleted and has a longer shelf life, which makes shopping easier and meal planning simpler. The product description is clear and understandable to me.*
topic_1	product, quick, fish, fresh,	Convenience	0.86	17	*The first thing that came to mind is that it is adapted to a busy lifestyle because it is clean and quick to prepare, which is certainly a plus for anyone who wants a freshly prepared lunch when they get home from work but do not want to "spend" a lot of time preparing it.*
topic_0	product, buy, price, prepare	Price	0.74	19	*I would like to buy a product that does not take me a lot of time to clean and prepare and does involve a lot of packaging waste. Due to work and hectic schedules during the week, I would definitely opt for such a product. I do mind the price - especially if I do have to bother with cleaning the fish.*
topic_2	product, fish, sardines, buy	Willingness to buy	0.73	20	*Sardines are a particularly dear fish to me, and we often eat them in the summer. Preferably on the grill. This type of package is suitable for daily consumption and especially for the winter season. I would buy it.*
topic_4	healthy, especially, eat, hurry	Healthiness	0.71	7	*It is especially suitable if you are in a hurry and do not want to eat something unhealthy.*
**Total chat texts**				**76**	

Table S7 Theme 3 Topics and sentiment scores_ ITALY

Topic id	Key Terms	Topic label	Overall sentiment	Number of chat texts	Chat text
topic_2	product, raw, fish, days	Eating raw	0.96	25	*I immediately thought about a very practical new product and even conservation, up to 30 days thanks to this method. The fact that you can eat it raw for the first few days impressed me, in general I never eat raw food but it's still a sign of quality.*
topic_5	product, fish, adjectives, prepare	Preparation mode	0.88	21	*I like the idea of the hamburger; I find it innovative and interesting. I would say that the adjectives that come to mind are innovative, practical and fast to prepare, certainly a good idea.*
topic_3	product, shrimp, dinner, family	Eating time	0.79	26	*Sounds like a good product to me. I would try it on occasion of a family dinner and experiment with new seasonings or combinations.*
topic_4	product, euros, price, dinner	Price	0.79	36	*I think the price could be around 8 euros. It can be eaten for both lunch and dinner, as it is immediately ready.*
topic_0	fish, raw, eat, fresh	Eating raw/fresh	0.78	27	*I do like raw fish but I never ate the cooked fish burger I would like to try. it seems like a good idea to me and in the fish departments I have not seen fresh fish burgers so I would like to try.*
**Total chat texts**				**156**	

Table S8 Theme 3 Topics and sentiment scores_ SPAIN

Topic id	Key Terms	Topic label	Overall sentiment	Number of chat texts	Chat text
topic_1	fish, prawns, eat, prefer	Species composition	0.82	69	*Regarding the combination of ingredients or species, I prefer as ingredients: prawns and mullet (also called smooth).*
topic_4	raw, fish, eat, cooked	Cooking	0.76	59	*I would do it cooked; I do trust to eat it raw. Maybe one day I will try it, but I think that once cooked it will have more flavor.*
topic_0	product, quality, similar, type	Product attributes perception	0.75	44	*On a scale up to 10, in principle I would give it a 7 until I see the type composition and quality.*
topic_2	raw, fish, product, consume	Eating raw	0.75	45	*I was positively surprised that it can be consumed raw considering how long is the period in which this can be done, up to 10 days!*
topic_3	meat, product, fish, flavor	Flavor expectation	0.73	53	*I already associated the hamburger with meat, it is true that it does not have meat, at least they did not put it, but it may have some flavor of meat.*
**Total chat texts**				**352**	

Table S9 Theme 3 Topics and sentiment scores_CROATIA

**Topic id**	**Key Terms**	**Topic label**	**Overall senti-ment**	**Number of chat texts**	**Chat text**
topic_5	product, eat, burger, try	Willingness to try	0.96	11	*A meat burger is a great thing, as is a veggie burger, so I see no reason why a fish burger should be less valuable. Of course, it should be combined with high quality foods. I would definitely buy and try such a product.*
topic_1	product, eat, fish, raw	Eating raw	0.90	10	*I like eating raw, but only under conditions where I know how much time has passed from catch to plate. I would only eat if it were caught one day ago.*
topic_3	fish, burger, prod-uct, eat	Eating	0.83	15	*I would prepare it when I wanted to eat something fishy unless I could buy something at the fish market. I'd also eat it as an addition to a fish menu, on the grill. I would love to see this product on our shelves.*
topic_2	product, buy, price, HRK	Price	0.79	23	*It is very likely that I will buy this product, at least I will definitely try it and the taste will decide another purchase. The price could be around 25-30 HRK.*
topic_0	product, raw, time, packaging	Preparation time	0.73	8	*I like product like this. Seafood, a healthy product that stays fresh longer. It can be eaten raw or prepared in other ways, with a short preparation time.*
**Total chat texts**				**67**	

Table S10 Theme 1 Emotion results with % value and absolute value_ALL COUNTRIES

	**Cooking**		**Habitual consumption**		**Sustainable fish and fish-ing**		**Freshness**		**Sustainable packaging**	
Emotions	*% on texts*	*emotion_count*	*% on texts*	*emotion_count*	*% on texts*	*emotion_count*	*% on texts*	*emotion_count*	*% on texts*	*emotion_count*
anger	43.1%	50	13.3%	33	15.7%	29	4.8%	16	10.4%	17
anticipation	100.0%	116	115.7%	288	49.2%	91	22.9%	76	38.0%	62
disgust	62.9%	73	20.5%	51	10.8%	20	5.4%	18	13.5%	22
fear	28.4%	33	19.3%	48	27.6%	51	6.3%	21	16.0%	26
joy	87.9%	102	82.7%	206	38.4%	71	18.7%	62	38.0%	62
sadness	32.8%	38	29.3%	73	18.9%	35	7.5%	25	12.9%	21
surprise	38.8%	45	21.3%	53	15.7%	29	11.1%	37	15.3%	25
trust	106.9%	124	87.6%	218	120.5%	223	52.4%	174	68.7%	112

Table S11 Theme 2 Emotion results with % value and absolute value_ ALL COUNTRIES

	**Convenience**		**Healthiness**		**Price**		**Species**	
Emotions	*% on texts*	*emotion_count*	*% on texts*	*emotion_count*	*% on texts*	*emotion_count*	*% on texts*	*emotion_count*
anger	5.30%	7	7.00%	9	10.10%	18	18.20%	20
anticipation	56.40%	75	150.80%	193	115.70%	206	50.90%	56
disgust	11.30%	15	17.20%	22	8.40%	15	10.90%	12
fear	10.50%	14	14.10%	18	14.60%	26	20.00%	22
joy	49.60%	66	99.20%	127	57.30%	102	48.20%	53
sadness	18.00%	24	32.00%	41	18.50%	33	20.00%	22
surprise	28.60%	38	29.70%	38	37.10%	66	24.50%	27
trust	84.20%	112	136.70%	175	75.30%	134	85.50%	94

Table S12 Theme 3 Emotion results with % value and absolute value_ALL COUNTRIES

	**Species**		**Eating time**		**Raw fish**		**Willingness to buy/eat**	
Emotions	*% on texts*	*emotion_count*	*% on texts*	*emotion_count*	*% on texts*	*emotion_count*	*% on texts*	*emotion_count*
anger	5.50%	7	12.70%	22	2.60%	3	7.00%	6
anticipation	29.70%	38	72.80%	126	91.20%	104	130.20%	112
disgust	8.60%	11	7.50%	13	8.80%	10	15.10%	13
fear	16.40%	21	16.20%	28	11.40%	13	18.60%	16
joy	27.30%	35	43.40%	75	78.90%	90	94.20%	81
sadness	14.10%	18	22.50%	39	12.30%	14	20.90%	18
surprise	17.20%	22	34.70%	60	43.00%	49	45.30%	39
trust	53.10%	68	67.60%	117	99.10%	113	104.70%	90
